# A real-world data analysis of montelukast in FDA Adverse Event Reporting System (FAERS) database

**DOI:** 10.1186/s40360-025-00959-3

**Published:** 2025-07-01

**Authors:** Hesen Huang, Kaiqin Chen, Yu Du, Jing Gao, Yixian Ye, Wei Lin

**Affiliations:** 1 Department of Otolaryngology-Head and Neck Surgery, Xiang’an Hospital of Xiamen University, Xia Men, Fu Jian 361102 China; 2 Department of Neurosurgery, Xiang’an Hospital of Xiamen University, Xia Men, Fu Jian 361100 China; 3 Department of Otolaryngology, Zhongshan Hospital (Xiamen), Fudan University, Xia Men, Fu Jian 361006 China; 4 Department of Otolaryngology-Head and Neck Surgery, The First Affiliated Hospital, Fujian Medical University, FuZhou, 350005 China; 5 Department of Otolaryngology-Head and Neck Surgery, National Regional Medical center, Binhai Campus of the First Affiliated Hospital, Fujian Medical University, Fuzhou, 350212 China

**Keywords:** MTK, Real-world data analysis, Adverse events, Pharmacovigilance, Disproportionality analysis

## Abstract

**Background:**

Montelukast(MTK) is a leukotriene receptor antagonist widely used clinically for treating asthma and rhinitis. However, many adverse events (AEs) have been reported. In this study, we aimed to investigate MTK’s adverse drug reactions (ADRs) using real data from the U.S. Food and Drug Administration Adverse Event Reporting System (FAERS) database.

**Methods:**

We assessed the disproportionality of MTK-associated AEs by calculating metrics such as the ratio of reported ratios (ROR), proportional reporting ratios (PRR), Bayesian Belief Propagation Neural Networks (BCPNN), and Gamma-Poisson Shrinkers (GPS).

**Results:**

From 2004 to the third quarter of 2023, of the 20,340,254 case reports in the FAERS database, 86,732 MTK reports were recorded as “principal suspect (PS)” AEs.Disproportionate analyses identified 431 preferred terms (PTs) associated with MTK in 27 organ systems. Unexpected major AEs were noted, such as Suffocation feeling, Adrenal suppression, Sudden visual loss and Endocardial fibrosis, none of which were mentioned in the drug insert.

**Conclusion:**

Our findings are consistent with clinical observations highlighting potential new and unexpected ADR signals associated with MTK. Further prospective clinical studies are needed to confirm and elucidate the relationship between MTK and these ADRs. This study provides a fresh and unique perspective on the study of adverse drug events.

**Supplementary Information:**

The online version contains supplementary material available at 10.1186/s40360-025-00959-3.

## Introduction

Leukocytes secrete substances known as leukotrienes (LTs), which function via three conjugated double bonds in the airway [1]. In 1980, LTC4 and LTD4 were shown to exhibit significant constrictor activity on isolated human bronchi [2]. This discovery led to an increase in subsequent studies examining LTs as potential treatments for asthma. Cysteinyl leukotriene receptors (CysLTRs) play a crucial role in regulating immune responses and are implicated in the pathophysiology of numerous respiratory allergic diseases, including bronchial asthma, exercise-induced asthma, aspirin-induced asthma, allergic rhinitis, atopic dermatitis, allergic conjunctivitis, and anaphylactic reactions [3–7]. Leukotriene receptor antagonists (LTRAs) and 5-lipoxygenase (5-LO) inhibitors are two classes of LT modifiers. MTK, zafirlukast, and pranlukast are LTRAs, while zileuton is a 5-LO inhibitor. Among these agents, MTK is the most frequently prescribed and extensively investigated globally [8]. 

MTK((1-([(1(R)-(3-(2-(7-chloro-2-quinolinyl)-(E)-ethenyl)phenyl)-3-(2-(1-hydroxy-1-methylethyl)phenyl)propyl)thio]methyl) is an antagonist of cysteinyl leukotriene receptor 1, which relaxes bronchial smooth muscle and has anti-inflammatory properties by regulating the bioactivity of leukotrienes. MTK is widely used to treat sleep-related breathing disorders, alleviate symptoms of allergic rhinitis, and prevent and treat asthma in both adult and pediatric patients [8–11]. It received its initial approval in the US and Europe in 1998 [12]. While MTK is generally well-tolerated, individual patient variability may lead to the development of unanticipated adverse events (AEs) in some individuals. Serious neuropsychiatric AEs have been documented in numerous clinical trials and post-marketing investigations [13–15]. The association between MTK and suicidal behavior has been confirmed through literature searches in International Drug Abstracts, MEDLINE, EMBASE, and the U.S. Food and Drug Administration (FDA) Adverse Event Reporting System (FAERS) [16]. This evidence prompted the FDA to issue multiple warnings regarding the increased risk of neuropsychiatric adverse events (AEs) associated with MTK and other leukotriene antagonists. These AEs include anxiety, depression, abnormal dreaming, euphoria, insomnia, hallucinations, irritability, aggressive behavior, and potential suicidality [11, 17, 18]. On March 4, 2020, the U.S. Food and Drug Administration (FDA) issued a Safety Bulletin calling for a Black Box Warning regarding serious neuropsychiatric adverse events (AEs) associated with MTK (Singulair) [11]. 

Adverse events (AEs) are occurrences that follow the use of medication. However, their presence does not automatically imply a causal relationship. Statistical, biological, or clinical analysis of these associations may, in some cases, reveal a causal link. When such a causal relationship is established, the event is referred to as an adverse drug reaction (ADR) [19]. 

Significantly, the FDA Adverse Drug Event Reporting System (FAERS), one of the largest pharmacovigilance databases globally, was established in 2012. It encompasses a wide range of adverse events (AEs) and medication errors associated with therapeutic biologics and drugs. The primary objective of FAERS is to monitor the safety of commercially available medications [20]. 

In this investigation, we employed disproportionality analyses to identify adverse drug reaction (ADR) signals associated with MTK in the FAERS database. The purpose of this investigation was to detect new and unexpected ADRs that are not listed on the drug label.

## Materials and methods

### Data sources and collection

FAERS is an open-access post-marketing safety surveillance database that compiles adverse event (AE) reports from specialized physicians, drug manufacturers, individual patients, and other sources. The DRUG data files in the FAERS database are downloaded using a combination of data cleaning tools and manual calibration techniques. The cleaning and mapping of drug names in FAERS are completed using OpenVigil 2 (https://openvigil.sourceforge.net Visit date: January 28, 2024)). In 2024, OpenVigil 2 utilized cleaned data to achieve more accurate results in pharmacovigilance data mining. Specifically, OpenVigil 2.1 provides FAERS with all relevant adverse events (AEs) reported for MTK Sodium. Data cleaning and standardization processes are managed using SAS and MySQL, which include merging data, removing duplicate records, applying standardized vocabulary, and aligning responses with the Medical Dictionary for Regulatory Activities (MedDRA), version 25.0 [21]. Adverse drug reactions (ADRs) related to MTK were compiled for a total of 79 quarters, spanning from the first quarter of 2004 to the third quarter of 2023. The MedDRA system was utilized to obtain the System Organ Class (SOC) and Preferred Term (PT), and to correct PT names in the FAERS database [22]. For this investigation, FAERS data were queried with MTK as the primary suspected drug. The classification of serious clinical outcomes included mortality (DE), disability (DS), hospitalization (HO), and life-threatening events (LT). The analysis also incorporated additional indicators such as gender, age, country, and reporter.

### Statistical analysis

Adverse event (AE) reporting of MTK using descriptive statistics. In our investigative study, disproportionality analysis, usually applied to pharmacovigilance studies, was performed to generate potential signals between MTK and adverse event response (ADR). Four main methods (i.e., Reported Ratio Ratio (ROR) [23], Proportional Reporting Ratio (PRR) [24], Bayesian Confidence Propagation Neural Network (BCPNN) [25], and Empirical Bayesian Geometric Mean (EBGM) [26] derived from Gamma-Poisson Shrinker (GPS) models) were used to assess the correlation between MTK and ADRs. The prerequisites are specified in Table 1, and detailed equations for the above methods are given in Table 2. We applied these four methods to detect FAERS signals from the first quarter of 2004 to the third quarter of 2023. Valid ADR results should fulfil the positive signal selection criteria of all four methods mentioned above at the same time. All MTK-related data processing and statistical analyses were performed using SAS, MySQL, WPS EXCEL and R software.

**Table 1:** 2 × 2matrix

	Target ADR	Non-target ADR
Drug of Interest	a	b
Other Drugs	c	d

Note: Montelukast is the target drug in this study, we retrieve montelukast data for analysis. N = a + b + c + d. ADR: adverse drug reaction

**Table 2:** Summary of algorithms

Algorithms	Equation^a^	Criteria
ROR	$\:ROR\:=\:\frac{(a/c)}{(b/d)}\:=\:\frac{ad}{bc}$	a ≥ 3, 95%CI (lower limit) > 1
	$$\:95\text{\%}\text{C}\text{I}={e}^{\text{l}\text{n}\left(\text{R}\text{O}\text{R}\right)\pm\:1.96\sqrt{(\frac{1}{a}\:+\:\frac{1}{b}+\frac{1}{c}+\frac{1}{d})}}$$	
PRR	$\text{PRR} = \frac{a/(a+b)}{c(c+d)}$	a ≥ 3, 95%CI (lower limit) > 1, PRR ≥ 2, χ2 ≥ 4
	$$\:95\text{\%}\text{C}\text{I}={e}^{\text{l}\text{n}\left(\text{P}\text{R}\text{R}\right)\pm\:1.96\sqrt{(\frac{1}{a}-\frac{1}{a+b}+\frac{1}{c}-\frac{1}{c+d})}}$$	
	$\:\chi\:$2=$\:\frac{(\text{a}\text{d}-\text{b}\text{c})^2\;(\text{a}+\text{b}+\text{c}+\text{d})}{(\text{a}+\text{b})(\text{a}+\text{c})(\text{c}+\text{d})(\text{b}+\text{d})}$	
BCPNN	IC = *log*_2_$\:\frac{p(x,y)}{p\left(x\right)p\left(y\right)}\:$= *log*_2_$\:\frac{a(a+b+c+d)}{(a+b)(a+c)}$	No signal (−):IC-2SD ≤ 0
	E (IC) = *log*_2_$\:\frac{(\text{a}+{\upgamma\:}11)(\text{a}+\text{b}+\text{c}+\text{d}+{\upalpha})(\text{a}+\text{b}+\text{c}+\text{d}+{\upbeta})}{(\text{a}+\text{b}+\text{c}+\text{d}+{\upgamma})(\text{a}+\text{b}+{\upalpha\:}1)(\text{a}+\text{c}+{\upbeta}1)}$	Weak signal (+):0 < IC-2SD ≤ 1.5
	V(IC) = $\:\frac{1}{\left(\text{l}\text{n}2\right)^2}${ [$\:\frac{\left(\text{a}+\text{b}+\text{c}+\text{d}\right)-\text{a}+{\upgamma\:}-{\upgamma}11}{\left(\text{a}+{\upgamma\:}11\right)\left(1+\text{a}+\text{b}+\text{c}+\text{d}+{\upgamma}\right)}$] + [$\:\:\frac{(\text{a}+\text{b}+\text{c}+\text{d})-(\text{a}+\text{b})+{\upalpha\:}-{\upalpha\:}1}{(\text{a}+\text{b}+{\upalpha\:}1)(1+\text{a}+\text{b}+\text{c}+\text{d}+{\upalpha})}$] $+\:[\:\frac{(\text{a}+\text{b}+\text{c}+\text{d})-(\text{a}+\text{c})+{\upbeta\:}-{\upbeta}1}{(\text{a}+\text{c}+{\upbeta}1)(1+\text{a}+\text{b}+\text{c}+\text{d}+{\upbeta})}]$}	Medium signal (++):1.5 < IC-2SD ≤ 3
	γ = γ11$\:\frac{(\text{a}+\text{b}+\text{c}+\text{d}+{\upalpha})(\text{a}+\text{b}+\text{c}+\text{d}+{\upbeta})\:}{(\text{a}+\text{b}+{\upalpha}1)(\text{a}+\text{c}+{\upbeta}1)}$	Strong signal (+++):IC-2SD > 3
	IC-2SD = E(IC)-2$\:\sqrt{\text{V}\left(\text{I}\text{C}\right)}$	
	p. s. α1 = β1 = 1; α = β = 2; γ11 = 1	
MGPS	EBGM = $\:\frac{\text{a}(\text{a}+\text{b}+\text{c}+\text{d})\:}{\:(\text{a}+\text{c})(\text{a}+\text{b})}$	EBGM05 > 2
	$$\:95\text{\%}\text{C}\text{I}={e}^{\text{l}\text{n}\left(\text{E}\text{B}\text{G}\text{M}\right)\pm\:1.96\sqrt{(\frac{1}{a}\:+\:\frac{1}{b}+\frac{1}{c}+\frac{1}{d})}}$$	

^a^ROR, reporting odds ratio; a, number of reports containing both the suspect drug and the suspect adverse drug reaction; b, number of reports containing the suspect adverse drug reaction with other medications (except the drug of interest); c, number of reports containing the suspect drug with other adverse drug reactions (except the event of interest); d, number of reports containing other medications and other adverse drug reactions; CI, confidence interval; PRR, proportional reporting ratio; χ2, chi-square; BCPNN, Bayesian confidence propagation neural network; IC, information component; IC-2SD, the lower confidence interval of IC; MGPS, multi-item gamma Poisson shrinker; EBGM, empirical Bayesian geometric mean; EBGM05, the lower 95% onesided CI of EBGM.

## Results

### General characteristics

**Fig. 1:**
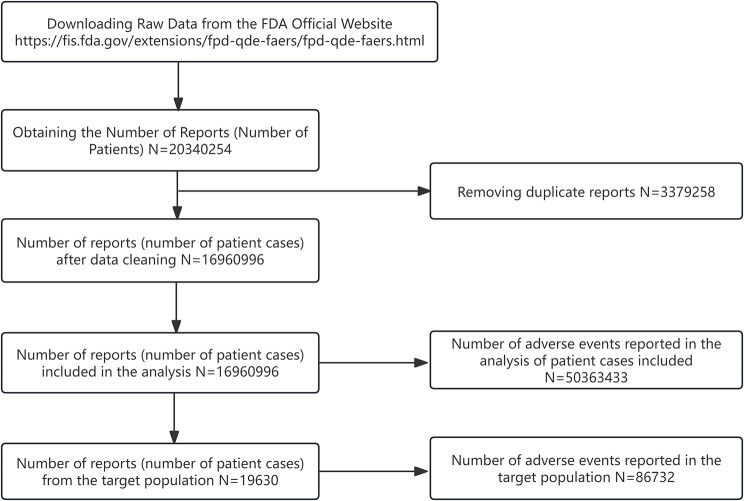
Process of selecting cases of montelukast-related AEs from the FAERS database

**Fig. 2:**
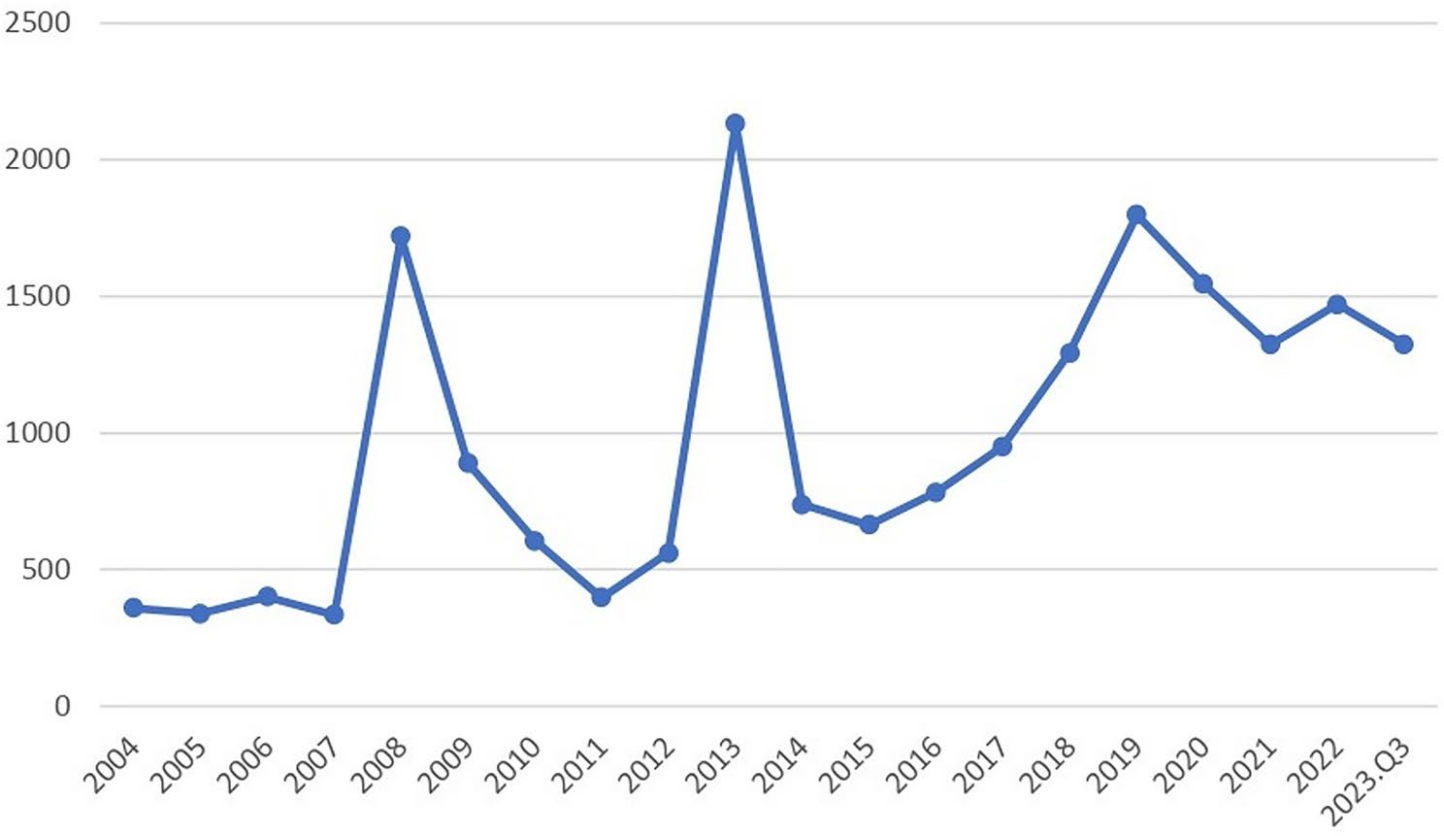
Annual trends in montelukast reporting

In Figs. 1, 20 and 340 case reports were collected from FAERS sources during the study period (January 2004 through September 2023). After excluding duplicates, 86,732 AEs associated with MTK as a PS drug were identified in 16,960,996 case reports. Detailed clinical characteristics of MTK events are described in Table 3 and Supplemental Fale. Among all AEs, in terms of sex ratio, males (53.01%) and females (37.33%). Regarding age composition, the lower age group under 18 years was more likely to experience AEs than other age groups with 31.01% (*n* = 6088). In addition, the top countries for MTK consumption were the United States (*n* = 10228, 52.10%), the United Kingdom (*n* = 3560, 18.14%), Canada (*n* = 1707, 8.70%), France (*n* = 486, 2.48%), and Japan (*n* = 384, 1.96%). The most common outcome of AEs is hospitalization (*n* = 3987, 20.31%), compared with the other three outcomes (death: *n* = 497, 2.53%; life-threatening: *n* = 1378, 7.02% and disability: *n* = 2207, 11.24%). Among the REPORTERS, Consumers had the highest percentage (*n* = 7702, 39.24%), followed by Physicians (*n* = 4565, 23.26%), Other health-professional (*n* = 3540, 18.03%), Pharmacists (*n* = 2316. 11.80%), and the lowest was Lawyer at 1.46% (*n* = 286). Considering the first quarter of 2004 through the third quarter of 2023 (Fig. 2), the highest year reported was 2013 (*n* = 2132, 10.86%).

**Table 3:** Characteristics of Montelukast related reports from January 2004 to September 2023.Q3

	Montelukast	
	Counts; *n*	Percentages; %
**Number of Events**	19,630	
**Gender**		
Male	10,405	53.01
Female	7327	37.33
Not Specified	1898	9.67
**Age**		
<18	6088	31.01
18–44	3104	15.81
45–64	3180	16.20
65–74	1259	6.41
75≤	699	3.56
NotSpecified	5300	27.00
**Reported Countries (the top ranked)**		
United States of America	10,228	52.10
United Kingdom	3560	18.14
Canada	1707	8.70
France	486	2.48
Japan	384	1.96
** Serious Outcomes**		
Life-Threatening	1378	7.02
Hospitalization	3987	20.31
Disability	2207	11.24
Death	497	2.53
** Reporter**		
Consumer	7702	39.24
Lawyer	286	1.46
Physician	4565	23.26
Other health-professional	3540	18.03
Pharmacist	2316	11.80
Not Specified	1221	6.22

### Signal detection

Table 4 displays the signal intensity of MTK at the SOC level and depicted MTK-induced AEs targeted 27 organ systems. Significant SOCs that met the criteria for at least one of the four indices were psychiatric disorders (SOC: 10037175, *n* = 30498), respiratory, thoracic and mediastinal disorders (SOC: 10038738, *n* = 8373), immune system disorders (SOC: 10021428, *n* = 1875) and social circumstances (SOC: 10041244, *n* = 676). We found that the social environment in the SOC mentioned above is a new and valuable ADR not listed in the drug instructions for MTK, while the other three items are mentioned in the instructions.

**Table 4:** Signal values of reports associated with Montelukast at the SOC level

System Organ Class	Case Reports	ROR(95% CI)	PRR(χ2)	IC(IC025)	EBGM(EBGM05)
Psychiatric disorders^a^	30,498	9.02(8.89, 9.15)	6.20(139583.55)	2.62(2.60)	6.14(6.06)
General disorders and administration site conditions	10,012	0.62(0.61, 0.63)	0.66(2089.33)	-0.59(-0.62)	0.66(0.65)
Respiratory, thoracic and mediastinal disorders^a^	8373	2.17(2.12, 2.22)	2.05(4740.62)	1.04(1.00)	2.05(2.01)
Nervous system disorders	7338	0.98(0.96, 1.00)	0.98(2.43)	-0.03(-0.06)	0.98(0.96)
Gastrointestinal disorders	4357	0.57(0.55, 0.58)	0.59(1376.67)	-0.77(-0.81)	0.59(0.57)
Injury, poisoning and procedural complications	3592	0.39(0.38, 0.40)	0.41(3301.82)	-1.27(-1.32)	0.41(0.40)
Skin and subcutaneous tissue disorders	3511	0.74(0.72,0.77)	0.75(301.05)	-0.41(-0.46)	0.75(0.73)
Investigations	3144	0.57(0.55,0.59)	0.58(990.18)	-0.77(-0.83)	0.58(0.56)
Infections and infestations	2638	0.57(0.55,0.60)	0.59(811.16)	-0.77(-0.83)	0.59(0.56)
Musculoskeletal and connective tissue disorders	2537	0.55(0.53,0.57)	0.56(915.61)	-0.83(-0.89)	0.56(0.54)
Immune system disorders^a^	1875	2.01(1.92,2.10)	1.98(920.98)	0.99(0.92)	1.98(1.89)
Cardiac disorders	1190	0.50(0.47,0.53)	0.51(583.23)	-0.98(-1.06)	0.51(0.48)
Eye disorders	921	0.53(0.50,0.57)	0.54(376.77)	-0.90(-0.99)	0.54(0.50)
Vascular disorders	903	0.47(0.44,0.51)	0.48(519.25)	-1.06(-1.15)	0.48(0.45)
Metabolism and nutrition disorders	829	0.43(0.40, 0.46)	0.44(613.71)	-1.19(-1.29)	0.44(0.41)
Product issues	786	0.58(0.54,062)	0.58(239.57)	-0.78(-0.88)	0.58(0.54)
Social circumstances^a^	676	1.69(1.57,1.83)	1.69(190.21)	0.75(0.64)	1.69(1.56)
Blood and lymphatic system disorders	585	0.40(0.37,0.43)	0.40(529.53)	-1.31(-1.43)	0.40(0.37)
Hepatobiliary disorders	520	0.65(0.60,0.71)	0.65(95.53)	-0.61(-0.74)	0.66(0.60)
Renal and urinary disorders	493	0.29(0.26,0.31)	0.29(861.03)	-1.77(-1.90)	0.29(0.27)
Surgical and medical procedures	391	0.34(0.31,0.38)	0.34(492.80)	-1.53(-1.68)	0.35(0.31)
Pregnancy, puerperium and perinatal conditions	355	0.93(0.84,1.03)	0.93(1.86)	-0.10(-0.26)	0.93(0.84)
Ear and labyrinth disorders	351	0.93(0.83,1.03)	0.93(2.03)	-0.11(-0.26)	0.93(0.83)
Neoplasms benign, malignant and unspecified (incl cysts and polyps)	253	0.10(0.09,0.12)	0.11(1926.98)	-3.22(-3.39)	0.11(0.10)
Congenital, familial and genetic disorders	230	0.86(0.75,0.98)	0.86(5.48)	-0.22(-0.41)	0.86(0.75)
Reproductive system and breast disorders	220	0.27(0.24,0.31)	0.28(421.71)	-1.86(-2.05)	0.28(0.24)
Endocrine disorders	154	0.71(0.61,0.83)	0.71(17.84)	-0.49(-0.72)	0.71(0.61)

^a^indicates statistically significant signals in the algorithm; ROR, reporting odds ratio; CI, confidence interval; PRR, proportional reporting ratio; χ2, chi-square; IC, information component; IC025, the lower limit of 95% CI of the IC; EBGM, empirical Bayesian geometric mean; EBGM05, the lower limit of 95% CI of EBGM

For AEs at the PT level, overall PT statistics for the 431 ADRs that met all four screening methods simultaneously are included in Supplemental Fale, and the top 50 PTs for MTK, ranked in descending order by ROR value, are shown in Table 5. The full 5 PTs were eosinophilic granulomatosis with polyangiitis (PT: 10078117, *n* = 743, IC025 = 7.31), neuropsychological symptoms (PT: 10089746, *n* = 428, IC025 = 7.03), tenon’s cyst (PT: 10066418 *n* = 10, IC025 = 2.32), separation anxiety disorder (PT: 10040045, *n* = 39, IC025 = 4.56), sleep terror (PT: 10041010, *n* = 659, IC025 = 6.54). In addition, in conjunction with Supplemental Fale, the top 4 MTK PTs by number of cases were anxiety (PT: 10002855, *n* = 2673, IC025 = 2.62), depression (PT: 10012378, *n* = 2371, IC025 = 2.74), suicidal ideation (PT: 10042458, *n* = 2007, IC025 = 3.85) and aggression (PT: 10001488, *n* = 1816, IC025 = 4.51). Notably, several unexpectedly significant AEs were identified that were not labelled in the labelling, including suffocation feeling (PT: 10042444, *n* = 17, IC025 = 1.30), adrenal suppression (PT:10001382, *n* = 12, IC025 = 1.77), sudden visual loss (PT:10042441, *n* = 9, IC025 = 1.91) and endocardial fibrosis (PT: 10014664, *n* = 3, IC025 = 0.38). Other unexpected PTs have been marked with an asterisk in Supplemental Fale.

**Table 5:** Ranked in descending order of adverse reaction signal intensity (top 50PT)

System organ class(SOC)	Preferred terms(PT)	Case Reports	ROR(95% CI)	PRR(Chi-Square)	IC(IC025)	EBGM(EBGM05)
Immune system disorders	Eosinophilic granulomatosis with polyangiitis	743	369.71(337.18-405.39)	366.55(165943)	7.43(7.31)	224.94(205.15)
Psychiatric disorders	Neuropsychological symptoms	428	358.23(317.54-404.14)	356.47(93946.0)	7.19(7.03)	221.11(196.00)
Injury, poisoning and procedural complications	Tenon’s cyst	10	724.68(286.00-1836.22)	724.60(3211.56)	3.41(2.32)	322.60(127.32)
Psychiatric disorders	Separation anxiety disorder	39	418.84(277.43-632.34)	418.66(9435.33)	5.11(4.56)	243.51(161.29)
Psychiatric disorders	Sleep terror	659	151.90(139.39-165.54)	150.75(77804.7)	6.67(6.54)	119.85(109.97)
Psychiatric disorders	School refusal	38	174.90(121.68-251.39)	174.82(5045.79)	4.92(4.41)	134.55(93.61)
Cardiac disorders	Endocarditis fibroplastica	8	272.82(117.73-632.17)	272.79(1473.12)	3.11(1.99)	185.82(80.19)
Gastrointestinal disorders	Sialadenosis	3	579.70(117.00-2872.26)	579.68(866.52)	1.98(0.17)	290.34(58.60)
Infections and infestations	Blebitis	10	207.05(100.57-426.26)	207.03(1510.77)	3.37(2.38)	152.81(74.23)
Respiratory, thoracic and mediastinal disorders	Sputum decreased	9	193.25(90.88-410.92)	193.23(1290.81)	3.23(2.20)	145.17(68.27)
Psychiatric disorders	Nocturnal fear	22	141.73(88.92-225.92)	141.70(2469.81)	4.27(3.61)	114.06(71.56)
Psychiatric disorders	Personality disorder of childhood	4	289.85(87.28–962.60)	289.84(767.58)	2.29(0.77)	193.56(58.28)
Psychiatric disorders	Obsessive-compulsive symptom	41	115.43(82.55–161.40)	115.37(3877.03)	4.88(4.40)	96.39(68.93)
General disorders and administration site conditions	Thumb sucking	4	257.65(79.34-836.66)	257.64(707.92)	2.29(0.78)	178.67(55.02)
Psychiatric disorders	Autophobia	30	100.56(68.24-148.18)	100.52(2519.14)	4.52(3.96)	85.81(58.24)
Investigations	Total lung capacity	3	248.44(64.24-960.79)	248.43(517.52)	1.97(0.29)	174.20(45.05)
Social circumstances	Fight in school	12	117.92(63.39-219.36)	117.90(1155.83)	3.53(2.66)	98.14(52.76)
Immune system disorders	Allergy to fermented products	4	178.37(58.16-547.06)	178.36(539.48)	2.28(0.82)	136.63(44.55)
Psychiatric disorders	Defiant behaviour	21	81.72(51.75-129.05)	81.70(1467.17)	4.09(3.43)	71.73(45.42)
Immune system disorders	Cockroach allergy	6	115.94(48.26-278.57)	115.94(569.72)	2.72(1.53)	96.78(40.28)
Investigations	Platelet adhesiveness decreased	3	173.91(47.86-631.93)	173.90(396.71)	1.97(0.33)	134.00(36.88)
Psychiatric disorders	Onychophagia	26	70.78(47.10-106.36)	70.76(1593.59)	4.26(3.67)	63.17(42.04)
Psychiatric disorders	Oppositional defiant disorder	36	65.65(46.51–92.67)	65.62(2058.04)	4.52(4.02)	59.05(41.83)
Psychiatric disorders	Paediatric acute-onset neuropsychiatric syndrome	6	105.40(44.16-251.56)	105.40(524.98)	2.71(1.53)	89.34(37.43)
Psychiatric disorders	Alice in wonderland syndrome	10	85.26(43.90-165.59)	85.25(725.85)	3.28(2.35)	74.45(38.33)
Investigations	Blood immunoglobulin E	4	122.04(41.52-358.75)	122.04(396.67)	2.26(0.85)	100.99(34.35)
Eye disorders	Pigment dispersion syndrome	10	79.42(41.01–153.80)	79.41(680.93)	3.26(2.34)	69.96(36.13)
Congenital, familial and genetic disorders	CFTR gene mutation	3	144.92(40.90-513.58)	144.92(343.02)	1.96(0.35)	116.14(32.77)
Social circumstances	Educational problem	120	49.16(40.80-59.23)	49.09(5212.10)	5.05(4.78)	45.34(37.62)
Psychiatric disorders	Tic	333	43.88(39.24–49.06)	43.71(12924.5)	5.19(5.02)	40.72(36.42)
Psychiatric disorders	Morbid thoughts	80	47.56(37.86–59.74)	47.51(3366.95)	4.84(4.51)	43.99(35.02)
Nervous system disorders	Sensory overload	10	72.47(37.55-139.85)	72.46(626.43)	3.25(2.33)	64.52(33.43)
Psychiatric disorders	Hyperarousal	5	96.62(37.49-249.03)	96.61(405.53)	2.50(1.23)	82.95(32.18)
Psychiatric disorders	Dysania	4	105.40(36.32-305.87)	105.40(349.99)	2.26(0.85)	89.34(30.78)
Investigations	Peak expiratory flow rate abnormal	8	72.47(34.75-151.13)	72.46(501.15)	3.00(1.98)	64.52(30.94)
Respiratory, thoracic and mediastinal disorders	Eosinophilic pneumonia chronic	19	52.96(33.11–84.72)	52.95(887.38)	3.84(3.17)	48.60(30.38)
Skin and subcutaneous tissue disorders	Chronic spontaneous urticaria	39	41.42(29.93–57.33)	41.41(1435.26)	4.32(3.84)	38.71(27.97)
Nervous system disorders	Mononeuropathy multiplex	25	43.79(29.16–65.77)	43.78(971.74)	4.01(3.42)	40.78(27.15)
Social circumstances	Physical abuse	13	51.27(29.08–90.41)	51.26(588.63)	3.45(2.65)	47.18(26.76)
General disorders and administration site conditions	Screaming	283	32.66(28.97–36.82)	32.56(8196.59)	4.80(4.63)	30.88(27.39)
Skin and subcutaneous tissue disorders	Nodular vasculitis	5	72.46(28.60-183.61)	72.46(313.22)	2.47(1.22)	64.52(25.46)
Psychiatric disorders	Nightmare	1339	27.80(26.30-29.38)	27.38(32521.4)	4.68(4.60)	26.19(24.79)
Congenital, familial and genetic disorders	Tourette’s disorder	41	35.38(25.81–48.50)	35.37(1290.50)	4.24(3.78)	33.39(24.36)
Psychiatric disorders	Attention-seeking behaviour	11	46.89(25.37–86.68)	46.89(457.02)	3.26(2.39)	43.45(23.51)
Blood and lymphatic system disorders	Hypereosinophilic syndrome	22	38.65(25.10-59.52)	38.65(756.35)	3.84(3.22)	36.29(23.57)
Psychiatric disorders	Aggression	1816	25.71(24.52–26.96)	25.20(40473.7)	4.58(4.51)	24.19(23.07)
Psychiatric disorders	Egocentrism	3	79.05(23.66-264.12)	79.05(203.44)	1.94(0.39)	69.68(20.86)
Psychiatric disorders	Obsessive-compulsive disorder	307	26.14(23.31–29.31)	26.05(7077.60)	4.53(4.37)	24.97(22.27)
Psychiatric disorders	Self esteem decreased	103	27.88(22.88–33.98)	27.85(2543.98)	4.42(4.13)	26.62(21.84)
Investigations	FEV1/FVC ratio decreased	6	52.70(22.85-121.56)	52.70(278.94)	2.64(1.49)	48.39(20.98)

^a^indicates statistically significant signals in the algorithm; ROR, reporting odds ratio; CI, confidence interval; PRR, proportional reporting ratio; χ2, chi-square; IC, information component; IC025, the lower limit of 95% CI of the IC; EBGM, empirical Bayesian geometric mean; EBGM05, the lower limit of 95% CI of EBGM

## Discussion

In general, most drugs’ efficacy and safety data originate from preclinical and clinical trials [27]. However, it can be challenging to fully understand how medications affect people in real-world settings, particularly regarding safety. This difficulty arises due to factors such as trial design and limited sample sizes. Therefore, to assess a medication’s safety and balance its benefits against risks in clinical decision-making, it is essential to continuously monitor risk signals of adverse drug reactions (ADRs) in clinical practice and report suspected drug-related events after a medicine has been launched [19]. To promote clinical pharmaceutical safety, pharmacovigilance in this study gathered and assessed a substantial amount of real-world data on the safety of MTK.

The FAERS database has been recognized as a valuable tool in pharmacovigilance for assessing drug safety in real-world settings. It offers several advantages, including broad monitoring coverage, representativeness, rapid reporting, and cost-effectiveness [28, 29]. From 2004 to the third quarter of 2023, the FAERS database collected 86,732 reports of adverse events (AEs) associated with MTK from various countries and territories worldwide. In terms of gender distribution, males accounted for 53.01% of the total reports, which was higher than females (37.33%). Regarding age, the proportion of AEs in the younger age group (< 18 years) was the highest at 31.01%, compared to other age groups. This finding is consistent with previous reports in the literature. For instance, in the VigiBase database, out of 972 MTK AE reports, 669 (69%) involved children under 18 years of age. Among these, 550 (57%) involved children between 2 and 10 years old. Additionally, males were more frequently reported among patients under 11 years of age, while females were more prevalent in reports involving patients aged 11 years and older [12]. This generally reflects the age- and gender-related prevalence of asthma, characterized by a higher prevalence in males before puberty and a higher prevalence in females after puberty. This shift is due to the increased incidence of asthma in females and the decreased remission rates after puberty [30]. Another study on the use of MTK in children reported a higher prevalence of adverse events (AEs) in 123 children (31.9%), with the highest prevalence among those aged 4–9 years (52.8%), followed by adolescents (24.4%) and toddlers (22.8%) [9]. The top two countries in terms of MTK consumption were the United States (10,228 reports, 52.10%) and the United Kingdom (3,560 reports, 18.14%). Among the reporters, consumers accounted for the highest proportion (*n* = 7,702, 39.24%), followed by physicians (23.26%), other health professionals (18.03%), and pharmacists (11.80%). The knowledge and attitudes of consumers and medical and health personnel are crucial, as educational interventions can significantly enhance the reporting of adverse events (AEs) in FAERS [31–33]. Therefore, increased outreach and educational efforts targeting FAERS databases should be considered to improve reporting by consumers and patients [34]. Many adverse events (AEs) may be underestimated if individuals or healthcare professionals do not take the time to report them to FAERS. A graph depicting annual trends in AE reporting from 2004 through the third quarter of 2023 (Fig. 2) shows peaks in MTK use in 2008, 2013, and 2019, followed by a gradual decline. The first two peaks may be related to sudden increases in respiratory illnesses in those years. The decline observed in late 2019 may be attributed to the strain on healthcare resources caused by the COVID-19 outbreak.

Significant signals at the System Organ Class (SOC) level, identified through imbalance analysis, included psychiatric disorders, respiratory, thoracic, and mediastinal disorders, and immune system disorders. Major adverse events (AEs) comprised neuropsychological symptoms, sleep terror, childhood personality disorder, Alice in Wonderland syndrome, nightmares, tics, nodular vasculitis, hypereosinophilic syndrome, chronic eosinophilic pneumonia, eosinophilic granulomatosis with polyangiitis, and chronic spontaneous urticaria. These findings are consistent with the AEs reported in the drug insert and clinical safety data [11, 15, 35]. We also identified several new and potentially unanticipated adverse events (AEs), such as choking sensation, adrenal suppression, sudden vision loss, and endocardial fibrosis. These findings are often difficult to detect in trials with limited populations. Ryogo Umetsu reported that common AEs associated with MTK use include upper respiratory tract infections, allergic reactions, diarrhea, nausea, vomiting, elevated liver enzyme levels, anxiety, irritability, depression, and sleep disturbances [11, 36]. Korean scholars have reported that the most common clinical adverse events (AEs) associated with MTK treatment include fever, upper respiratory tract infections, and worsening of asthma symptoms [1]. 

Eleonora Di Salvo reported that MTK can cause cutaneous adverse reactions in asthmatic patients, with approximately half of these patients experiencing symptoms such as rash, blisters, skin macules, purpura, cutaneous maculopapular rash, erythematous rash, urticaria, and eosinophilic granulomatous polyangiitis [37]. Eosinophilic granulomatous polyangiitis (EGPA), also known as Churg-Strauss syndrome (CSS), is a rare ANCA-associated vasculitis that can occur in patients with a history of asthma or allergic rhinitis. It was once considered a rare complication potentially caused by MTK [11, 14]. A case-crossover study involving 78 patients with CSS reported that MTK use was associated with a 4.5-fold increased risk of developing CSS within three months [38]. However, the treatment for CSS typically includes glucocorticoids and other immunosuppressive drugs [1]. The Reporting Odds Ratio (ROR) signaling for Eosinophilic Granulomatous Polyangiitis (EGPA) suggests an association with MTK use. However, EGPA is not caused by MTK itself, but rather by a reduced dose of glucocorticoids when used in combination with MTK. The elevated ROR values associated with EGPA are considered to be superficially higher [11]. It has been suggested that MTK may merely act as a confounding factor [1]. The Dutch pharmacovigilance database reported eight cases of allergic granulomatous vasculitis, while VigiBase reported 563 cases. Despite these reports, it remains unclear whether a causal relationship between MTK and Churg-Strauss syndrome (CSS) has been definitively established [15]. 

Chinese scholars have observed that MTK-related urogenital symptoms—such as enuresis, dyspareunia, and hematuria—are not uncommon in China but are less frequently reported internationally. These symptoms warrant serious attention [36]. In another study from Korea, when adverse drug reactions (ADRs) associated with MTK and Prilosec were categorized by system organ class, the most frequent ADRs were related to the gastrointestinal system, followed by psychiatric events. These adverse reactions were more prevalent in females. Therefore, scholars recommend that when prescribing MTK and Prilosec, clinicians should pay particular attention to gastrointestinal and sleep disorders, especially in females aged 19 to 64 [35]. 

Recently, the identification of new molecular targets has highlighted the importance of MTK’s systemic anti-inflammatory effects, particularly in brain tissue. Various clinical studies have focused on repurposing the drug for use in other disorders, especially Alzheimer’s disease and Parkinson’s disease. However, this repurposing has been challenged by the adverse neuropsychopharmacological effects associated with the drug [8]. MTK-induced adverse drug reactions (ADRs), particularly neurocognitive events, have been previously studied, primarily in children and in relation to suicidal ideation and behavior [12, 39]. In a prospective observational study, 78 out of 125 children (62.4%) who were initially prescribed MTK for asthma reported neuropsychiatric adverse effects that resolved upon discontinuation of the medication. The incidence of temperamental behavior, nightmares, and sleep disturbances was significantly higher in both groups compared to pre-treatment levels (*P* < 0.001). The frequency of neuropsychiatric adverse reactions caused by MTK was higher than previously reported in the literature, consistent with our findings. These neuropsychiatric adverse effects can have a significant negative impact on the quality of life of children [40]. As of May 3, 2020, the VigiBase database contained 25,120 post-marketing reports of suspected or interacting MTK, of which 32% reported psychiatric adverse reactions. This included 1,118 reports of nightmares, with approximately half classified as serious effects. Two-thirds of the reports involved children under 18 years of age, the majority of whom were aged 2–10 years, with the highest number of reports coming from the 5–10-year age group. In most cases, nightmares resolved upon discontinuation of the drug, but in some patients, they persisted for an extended period [41]. Another study on neuropsychiatric events associated with MTK—such as depression, aggression, suicidal ideation, aberrant behavior, and nightmares—found that these events were more prevalent in children compared to adults [39]. Among children aged 2–10 years, aggression was the most commonly co-reported adverse effect, while in adolescents and adults, anxiety and depression were more frequently reported. For patients aged 65 years and older, the most common co-reported terms were insomnia, headache, and muscle cramps. Clinicians should take these findings seriously when evaluating patients presenting with such behaviors and symptoms. Among 146 patients with co-reported suicidal and self-injurious behaviors, the highest percentage (35%) was observed in patients aged 11–17 years [12]. It was previously believed that MTK had difficulty crossing the blood-brain barrier. However, a recent update to the US label suggests that this may indeed be possible. The label now acknowledges that studies have demonstrated the drug’s delivery to the brains of rats. This finding suggests that MTK could similarly affect humans, thereby providing a potential explanation for the development of adverse central nervous system (CNS) reactions [42]. A multi-omics approach confirms that miglustat interferes with the glutathione detoxification system in the brain and can deregulate a wide variety of neurotransmitter and neurosteroid pathways. This impact is particularly pronounced in pathways involved in the hypothalamus-pituitary system, including those regulating the hypothalamic-pituitary-adrenal (HPA) axis and those interfering with mitochondrial function in neuronal cells. The therapeutic effects of MTK are accompanied by significant modulation of specific processes in the central nervous system, which may explain the associated neuropsychiatric responses. Additionally, the findings suggest that adverse drug reactions (ADRs) are more likely to occur in children due to their earlier stage of brain maturation [43]. Several reports have documented improvements in nightmares after changing the dosage regimen and switching to taking the medication in the morning rather than at night. The Mayo Clinic’s dosing recommendations offer patients the option of taking MTK either at night or in the morning, providing those who experience nightmares the possibility of trying an alternative dosing regimen.After reassessing the benefits and risks of MTK, the FDA strengthened the existing labeling to require a black box warning for serious behavioral and mood-related changes associated with MTK. The FDA also recommended limiting its use to allergic rhinitis [12]. 

Despite the advantages of real-world, large-scale population studies and the data-mining techniques employed, this research has several limitations. The FAERS database, which relies on spontaneous reporting, accepts reports from both healthcare professionals and non-professionals. While this inclusivity broadens the scope of information, it also introduces variability in report quality, potentially leading to biased evaluations (e.g., the Weber effect and notoriety effect). Additionally, controlling for confounding variables—such as dosage, duration of use, comorbidities, drug combinations, and other factors that may influence adverse events (AEs)—is a significant challenge. Furthermore, the precise incidence rate of each AE could not be determined due to the lack of data on the entire patient population using MTK in real-world settings. Lastly, this study did not establish a causal relationship between MTK and adverse drug reactions (ADRs). This is an important limitation, as the disproportionality analysis used only provides a statistically significant indication of signal strength, without quantifying risk or establishing causality. Disproportionate analysis only represents associations and cannot infer direct drug ADR relationships. However, compared to other existing studies, our research is uniquely supported by a substantial corpus of international data in quantifying potential risk. Nevertheless, prospective studies are required to determine the actual risk associated with these adverse drug reactions (ADRs).

Given its widespread and prevalent use, MTK is associated with adverse drug reactions (ADRs) that can affect various organs. However, it is important to note that the ADR precautions specified in the drug insert do not sufficiently address all potential ADRs. Consequently, medical personnel and consumers should be informed of any ADRs identified in this study that are not explicitly mentioned on the drug’s label.In conclusion, MTK is readily available as an over-the-counter (OTC) formulation, allowing individuals to obtain it without a prescription. This accessibility necessitates continued vigilance regarding the potential adverse effects of daily intake quantities.

## Conclusion

We conducted pharmacovigilance analyses using real-world data from the FAERS database to identify potential hazards and safety concerns associated with MTK. Our findings suggest that MTK may also cause unexpected serious adverse reactions, including endocardial fibrosis, sudden visual impairment, adrenal suppression, and asphyxia. Further prospective clinical studies are needed to validate and elucidate the correlation between MTK and these adverse drug reactions (ADRs). This study offers a novel and unique perspective for investigating adverse drug events.

## Electronic supplementary material

Below is the link to the electronic supplementary material.

Supplementary Material 1

## Data Availability

No datasets were generated or analysed during the current study.
